# Retraction Note to: Partially hydrolysed, prebiotic supplemented whey formula for the prevention of allergic manifestations in high risk infants: a multicentre double-blind randomised controlled trial

**DOI:** 10.1186/s13601-020-00356-5

**Published:** 2020-11-09

**Authors:** Robert Boyle, Nick Brown, Wen Chin Chiang, Chua Mei Chien, Michael Gold, Jonathan Hourihane, Jane Peake, Patrick Quinn, Raj Rao, Peter Smith, Mimi Tang, John Ziegler, John Warner

**Affiliations:** 1 Imperial College London, London, UK; 2 Salisbury Healthcare NHS Trust, Salisbury, UK; 3 KK Women’s and Children’s Hospital, Singapore, Singapore; 4 Women’s and Children’s Hospital, Adelaide, Australia; 5 University College, Cork, Ireland; 6 Royal Children’s Hospital, Brisbane, Australia; 7 Poole Hospital NHS Trust, Poole, UK; 8 Gold Coast Hospital, Gold Coast, Australia; 9 Murdoch Children’s Research Institute, Melbourne, Australia; 10 Sydney Children’s Hospital, Sydney, Australia

## Retraction Note to: Clin Transl Allergy (2015) 5(Suppl3):P30 10.1186/2045-7022-5-S3-P30

The authors have retracted this poster abstract [1] because of concerns regarding the validity of the post-hoc analysis presented here. An independent statistical analysis has found that the post-hoc analysis used a very small sample, which means the results are not generalizable to the original sample included in the trial. Conclusions based on results from this restricted sample are therefore unreliable. The effects seen are unlikely to be due to the active intervention and instead might be due to residual confounding related to age of weaning.

All authors agree to this retraction.
